# Prevalence and Associated Factors of Depression Among Emergency Physicians in South Korea: Findings from the 2025 Korean Emergency Physician Survey

**DOI:** 10.3390/medicina62030504

**Published:** 2026-03-09

**Authors:** Min Jae Kim, In Hwan Yeo, Mi Jin Lee, Ji Hun Kim, Hyung Min Lee, Kwang Hyun Cho, Kyung Hye Park, Eu Sun Lee, Joon Bum Park, Sanghun Kim, Ji Eun Kim, Han Zo Choi, Kyungseok Park

**Affiliations:** 1 Department of Emergency Medicine, Kyungpook National University Chilgok Hospital, School of Medicine, Kyungpook National University, Daegu 41944, Republic of Korea; minj9506@naver.com; 2 Department of Emergency Medicine, St. Vincent’s Hospital, College of Medicine, The Catholic University of Korea, Seoul 03083, Republic of Korea; emergency.mijinlee@gmail.com; 3 Department of Emergency Medicine, Uijeongbu St. Mary’s Hospital, College of Medicine, The Catholic University of Korea, Uijeongbu 11765, Republic of Korea; remind1580@naver.com; 4 Department of Emergency Medicine, Inje University Ilsan Paik Hospital, Goyang 10380, Republic of Korea; lee.hyungmin.em@gmail.com; 5 Department of Emergency Medicine, Eulji Medical Center, Eulji University, Seoul 01830, Republic of Korea; guskhan@eulji.ac.kr; 6 Department of Medical Education, Yonsei University Wonju College of Medicine, Wonju 26426, Republic of Korea; erdoc74@gmail.com; 7 Department of Preventive Medicine, Ulsan University College of Medicine, Seoul 05505, Republic of Korea; eusunlee@naver.com; 8 Department of Emergency Medicine, Soonchunhyang University College of Medicine, Seoul 04401, Republic of Korea; jesumania@gmail.com; 9 Department of Biomedical Engineering, Seoul National University College of Medicine, Seoul 03080, Republic of Korea; efu0211@snu.ac.kr; 10 Department of Emergency Medicine, College of Medicine, Dong-A University, Busan 49201, Republic of Korea; amcfsapple@dau.ac.kr; 11 Department of Emergency Medicine, Kyunghee University Hospital at KangDong, Seoul 05278, Republic of Korea; sacehan@daum.net; 12 Department of Emergency Medicine, Seoul Metropolitan Government-Seoul National University Boramae Medical Center, Seoul 07061, Republic of Korea

**Keywords:** emergency physicians, depression, workplace violence, sleep deprivation, burnout, professional, stress, psychological, Republic of Korea

## Abstract

*Background and Objectives*: Emergency physicians practice in high-pressure environments and face occupational stressors that may affect their mental health. This study was designed to evaluate the prevalence of depression among emergency physicians in South Korea and examined environmental, sociolegal, and individual factors associated with depressive symptoms in the post-pandemic period. *Materials and Methods*: This nationwide cross-sectional study analyzed data from the 2025 Korean Emergency Physician Survey. Screening positive for depressive symptoms was defined as a Patient Health Questionnaire-9 (PHQ-9) score ≥ 10, indicating moderate-to-severe depressive symptom severity. Measures included the PHQ-9, the Korean Epworth Sleepiness Scale (KESS), and the Adult APGAR, a brief self-administered instrument assessing overall wellness. Multivariable logistic regression was performed to identify factors associated with depression after adjusting for demographic, clinical, and work-related variables. *Results*: Of the 1050 physicians who responded (response rate: 37.5%), 743 emergency physicians completed the PHQ-9 section (completion rate: 70.8%; mean age, 43.2 ± 7.78 years; 86.5% male), and 111 (14.9%) screened positive for depressive symptoms. Objective workload indicators, including total work hours and number of night shifts, did not differ between physicians with and without depression. However, emergency physicians screening positive for depression reported higher perceived burdens related to staffing shortages and patient-related stressors. Protective factors included being married (adjusted odds ratio [AOR], 0.22; 95% confidence interval [CI], 0.08–0.58), longer sleep duration (AOR, 0.65; 95% CI, 0.50–0.86), better sleep quality (AOR, 0.45; 95% CI, 0.27–0.74), fixed mealtimes (AOR, 0.60; 95% CI, 0.39–0.93), and higher Adult APGAR scores (AOR, 0.72; 95% CI, 0.60–0.86). Factors associated with increased odds of depression included a history of cancer (AOR, 14.63, 95% CI, 2.53–84.61), current alcohol consumption (AOR, 2.54, 95% CI, 1.14–5.68), daytime sleepiness (AOR, 1.17; 95% CI, 1.04–1.31), and more frequent verbal abuse during the previous 12 months (AOR, 1.25; 95% CI, 1.08–1.44). *Conclusions*: Depression was prevalent and was associated with perceived work burden, sleep health, lifestyle regularity, and psychosocial factors. Interventions should address sleep quality, workplace safety, and social support.

## 1. Introduction

Depression constitutes a major public health challenge in modern society, causing not only psychological distress but also substantial socioeconomic loss. According to the Organization for Economic Co-operation and Development (OECD, 2021), South Korea reported a depression prevalence of 36.8%, the highest among surveyed member countries [1]. Statistics from the Korea Disease Control and Prevention Agency (KDCA) show that the proportion of the population experiencing depressive symptoms for 2 weeks or longer has steadily increased since 2018, reaching 7.7% in 2023 [2]. The number of patients receiving hospital treatment for depression in South Korea has also steadily risen, surpassing one million in 2023. The number of patients receiving outpatient care for depression in South Korea has also steadily risen, increasing by 36.8% from 2018 to 2023 and reaching 1,441,676 in 2023 [3].

The emergency medical system serves as the frontline of essential healthcare, providing 24 h service crucial for the treatment of patients with acute illnesses and trauma, and acting as a critical safety net for the community. Emergency physicians are constantly exposed to psychological pressure because they must make high-stakes decisions with limited information and time in urgent environments. This situation poses a substantial threat to their mental health, primarily due to disruption of the circadian rhythm caused by irregular shift work and night shifts, chronic sleep deprivation, and the psychological burden of encountering patients near death or experiencing unexpected clinical outcomes [4,5].

Factors contributing to depressive symptoms among emergency physicians can be categorized into environmental and socio-legal stressors. Environmental stressors include patient overcrowding and persistent understaffing, which may exacerbate depressive symptoms. Socio-legal stressors, such as the increasing rate of medical malpractice lawsuits and exposure to violence and verbal abuse within the emergency department (ED), also constitute major causes of depression among emergency physicians [6,7]. A previous study reported that emergency medicine specialists and residents in South Korea experience higher levels of burnout and depressive symptoms than their counterparts in other medical specialties [8].

Depressive symptoms among medical staff not only diminishes the individual physician’s quality of life but can also lead to impaired judgment and reduced concentration, thereby increasing the risk of medical errors and posing a direct threat to patient safety [9]. Furthermore, this situation may accelerate the avoidance of emergency medicine as a specialty and the attrition of skilled professionals, potentially leading to a breakdown of the national emergency medical system [10].

In South Korea, the Korean Emergency Physician Survey (KEPS) is conducted at five-year intervals, providing a valuable opportunity to assess the evolving status of emergency physicians and to monitor changes in working conditions and health-related outcomes over time. While the survey conducted five years ago captured emergency physicians’ experiences during the COVID-19 pandemic, the 2025 survey reflects a distinct but comparably challenging period marked by intense conflict between the medical community and the government and a large-scale disruption of the resident workforce. During this period, many tertiary hospital emergency departments faced severe staffing shortages, and remaining emergency medicine attending physicians were confronted with excessive workloads and heightened occupational stress [11].

In addition to these system-level changes, the 2025 KEPS expanded its scope compared with prior waves by including items on chronic health conditions and health status—such as a history of cancer, hypertension, diabetes, and obesity—enabling a more comprehensive evaluation of how physicians’ baseline health may interact with occupational stressors and affect depressive symptoms. This expanded measurement represents an important advancement over earlier studies that primarily focused on work environment factors alone.

Therefore, this study was designed to estimate the prevalence of screening positive for depressive symptoms and to identify the complex factors associated with depressive symptoms among emergency physicians in South Korea, using data from the 2025 KEPS. We hypothesized that excessive workload and sleep-related problems (e.g., insufficient sleep), as well as underlying medical conditions (e.g., cancer and other chronic diseases), would be associated with a higher likelihood of screening positive for depressive symptoms among emergency physicians.

## 2. Materials and Methods

### 2.1. Data Collection and Participants

This study utilized data from the 2025 KEPS [12]. Survey links were distributed via email and SMS to 2804 board-certified emergency physicians in Korea from 7 August to 12 September 2025. The survey covered seven domains; this analysis focused on working conditions, individual characteristics, and mental health. Because this study involved a retrospective analysis of previously collected survey data with minimal risk to participants, retrospective Institutional Review Board (IRB) approval was obtained.

### 2.2. Measures

Depression was assessed using the Korean version of the Patient Health Questionnaire-9 (PHQ-9), a validated self-report tool designed to screen for and monitor the severity of depressive symptoms [13,14]. The PHQ-9 consists of nine items corresponding to the Diagnostic and Statistical Manual of Mental Disorders, Fourth Edition (DSM-IV) diagnostic criteria for major depressive disorder. Respondents rated the frequency of symptoms over the past 2 weeks on a 4-point Likert scale, ranging from 0 (not at all) to 3 (nearly every day), yielding a total possible score ranging from 0 to 27. In this study, a total PHQ-9 score of ≥10 was used as the threshold for screening positive for depressive symptoms, indicating moderate-to-severe depressive symptom severity; this cutoff is widely recognized for its high sensitivity and specificity in clinical and epidemiological research [14].

Daytime sleepiness was assessed using the Korean Epworth Sleepiness Scale (KESS), which has demonstrated reliability and validity in the Korean population [15]. Each item was rated on a 3-point Likert scale from 0 (not at all) to 2 (very much) (i.e., “not at all”, “somewhat”, “quite a bit”, and “very much”), yielding a total possible score ranging from 0 to 16, with higher scores indicating greater daytime sleepiness.

Sleep duration was assessed as the self-reported average number of hours slept per day. Sleep quality and self-rated health were measured using 5-point Likert scales ranging from 1 (very poor) to 5 (very good), with higher scores indicating better perceived status.

Overall wellness was assessed using the Adult APGAR, a brief, proactive, self-administered 10-point (self-scoring) instrument that evaluates adult well-being across five domains: Access, Priorities, Growth, Assistance, and Responsibility. This instrument has been previously described for assessing physician wellness [16]. Items were rated on a 3-point Likert scale from 0 (“rarely”) to 2 (“always”) (i.e., “rarely”, “sometimes”, and “always”), and summed to generate a total score, yielding a total possible score ranging from 0 to 10, with higher scores indicating better perceived wellness and self-management, including aspects related to work–life balance and health management rather than physical symptoms alone.

Smoking and alcohol consumption were assessed using separate status and exposure measures. Participants first indicated whether they currently smoked (yes/no). Among current smokers, cumulative smoking exposure was quantified in pack-years. Participants were also asked whether they generally consumed alcohol (yes/no). Among those reporting alcohol use, drinking frequency was assessed as the number of drinking occasions per month. Status and exposure variables were analyzed separately.

Work environment perceptions—including schedule satisfaction, salary satisfaction, perceived emergency department safety, and perceived burdens related to staffing shortages and patient-/organization-related stressors—were assessed using 5-point Likert-type items ranging from 1 (strongly disagree) to 5 (strongly agree), with higher scores indicating greater agreement with each statement.

Workplace violence exposure was assessed using self-reported items that distinguished between personal exposure and department-level occurrence. Participants were asked whether they had directly experienced verbal abuse or physical assault during the past 12 months, and the frequency of these events was recorded. In addition, respondents reported the frequency of verbal abuse or physical assault occurring within their affiliated emergency department during the past month, regardless of whether they were personally targeted. Accordingly, individual-level exposure (past 12 months) and department-level occurrence (past 1 month) were operationalized as separate variables.

Current engagement in emergency department (ED) clinical practice was assessed using a binary item asking whether respondents were currently providing ED care (yes/no). Respondents who indicated that they were not currently engaged in ED practice were automatically skipped from subsequent items related to ED-specific workload and occupational stressors.

Marital status was assessed as a categorical variable (married, never married, divorced/separated, widowed, or non-response). For analytic purposes, this variable was dichotomized into married versus non-married (including never married, divorced/separated, and widowed). Professional position was assessed as a single-response item, and participants were instructed to select the one category that best reflected their primary current role.

Consistent with occupational stress and biopsychosocial models of depression, independent variables were categorized into demographic characteristics, health vulnerability factors, lifestyle and sleep-related factors, psychosocial resources, and workplace stress exposures, as detailed above.

In the present sample, internal consistency was good for all multi-item scales (PHQ-9: Cronbach’s α = 0.88, 95% CI 0.86–0.89; KESS: α = 0.84, 95% CI 0.82–0.85; Adult APGAR: α = 0.83, 95% CI 0.81–0.85).

### 2.3. Statistical Analysis

All statistical analyses were conducted using R version 4.5.2 (R Core Team, 2025; R Foundation for Statistical Computing, Vienna, Austria). Descriptive statistics and group comparisons were performed using the mytable() function from the moonbook package, which automatically applies appropriate statistical tests (e.g., chi-square test, Fisher’s exact test, *t*-test, or ANOVA) according to variable type. Continuous variables were expressed as mean ± standard deviation (SD). Normality of key continuous variables was assessed using visual inspection of Q–Q plots. Although some variables demonstrated mild skewness, particularly count-based measures, given the large sample size, parametric tests were considered robust to moderate deviations from normality. A *p*-value ˂ 0.05 was considered statistically significant.

To identify independent predictors of depression risk, a two-step variable selection process was employed. First, candidate variables were selected based on univariable screening using a liberal significance threshold of *p* < 0.25 or clinical relevance. Second, to address potential overfitting and multicollinearity among numerous predictors, least absolute shrinkage and selection operator (LASSO) logistic regression was performed. The optimal tuning parameter (λ) was determined using 10-fold cross-validation, and variables with non-zero coefficients at λmin were retained. Finally, a multivariable logistic regression model (non-penalized generalized linear model) was fitted using the LASSO-selected variables to estimate unbiased adjusted odds ratios (AORs) and their 95% confidence intervals. Following LASSO selection and prior to fitting the final model, multicollinearity diagnostics were assessed using variance inflation factors (VIFs). All retained predictors demonstrated VIF values below 2.1, indicating no evidence of problematic multicollinearity and supporting model stability. This post-selection inference approach was used to enhance model stability and reduce overfitting while identifying robust independent predictors.

## 3. Results

### 3.1. Baseline Characteristics

A total of 1050 physicians responded to the survey (response rate: 37.5%), and 743 of these respondents completed the PHQ-9 section, yielding a completion rate of 70.8%. Among these physicians (mean age, 43.2 ± 7.78 years; 86.5% male), 111 (14.9%) screened positive for depressive symptoms (PHQ-9 ≥ 10). No significant differences were observed between physicians with and without depression with respect to age or sex distribution (Table 1).

However, physicians screening positive for depression were significantly less likely to be married than their non-depressed counterparts (76.6% vs. 88.4%, *p* = 0.001). No significant differences were observed between groups in position type or in the proportion with a body mass index (BMI) ≥ 30, although BMI demonstrated a borderline association with screen-positive physicians (*p* = 0.052). In contrast, hypertension, arrhythmia, cardiovascular disease, and cancer were more prevalent among screen-positive physicians, whereas diabetes and cerebrovascular disease did not differ significantly between groups.

Physicians screening positive for depression reported lower exercise frequency than those without depression (1.9 ± 1.6 vs. 2.4 ± 1.7 times per week, *p* = 0.003). Although overall drinking prevalence did not differ significantly between groups, screen-positive physicians reported more frequent alcohol consumption per month (7.5 ± 5.7 vs. 6.0 ± 5.3, *p* = 0.035). Smoking prevalence did not differ significantly between groups, while smoking intensity showed a trend toward higher pack-year exposure among screen-positive physicians, without reaching statistical significance.

### 3.2. Work Environment, Workload, and Eating-Related Factors

Physicians screening positive for depression reported lower satisfaction with their work schedules, whereas salary satisfaction did not differ significantly between groups (Table 2).

Objective workload indicators, including the number of working days per month, number of night shifts, total working hours, and annual ED visit volume, did not differ significantly between physicians with and without depression. Staffing levels, including the number of emergency medicine specialists, residents, and interns, were also comparable between groups.

In contrast, most perceived workload and relational burden measures were significantly higher among physicians screening positive for depression. These included perceived work-hour burden and work intensity compared with other departments, perceived inadequacy of salary relative to work intensity, and multiple patient- and system-related stressors. These stressors included higher perceived burdens related to patient volume, critical care management, explaining clinical decisions to other departments, attitudes of other departments toward emergency medicine, transfer-out and transfer-in processes, admission requests and discharge refusals, relationships with tertiary hospitals, long-term care facilities, and public health authorities, as well as physician and nursing staff shortages (all *p* < 0.05). Perceived burden related to EMT shortages and security staffing did not differ significantly between groups.

Regarding eating-related factors, physicians screening positive for depression were less likely to have fixed mealtimes, less able to take meals despite ED workload, reported poorer access to regular hospital-provided meals, and perceived their mealtimes as less sufficient (all *p* ≤ 0.001). They were also more likely to consume meals during night shifts, while regular breakfast consumption did not differ significantly between groups. Finally, physicians screening positive for depression perceived their overall diet as substantially less healthy than their non-depressed counterparts.

### 3.3. Mental Health, Resilience, Violence, and Legal Issues

Physicians screening positive for depression had poorer self-rated health, shorter sleep duration, worse sleep quality, and higher Korean Epworth Sleepiness Scale scores compared with physicians without depression (all *p* < 0.05) (Table 3).

Adult APGAR scores, an instrument reflecting overall wellness status, were substantially lower in the depression group. Exposure to workplace violence was more frequent among physicians screening positive for depression, who reported higher numbers of verbal abuse incidents and physical assault episodes both over the past 12 months and during the past month. In addition, physicians screening positive for depression perceived the ED as less safe and were significantly more likely to have experienced recent legal disputes within the past year. In contrast, involvement in civil or criminal litigation showed only a borderline difference between groups.

### 3.4. Multivariable Analysis

**Figure 1:**
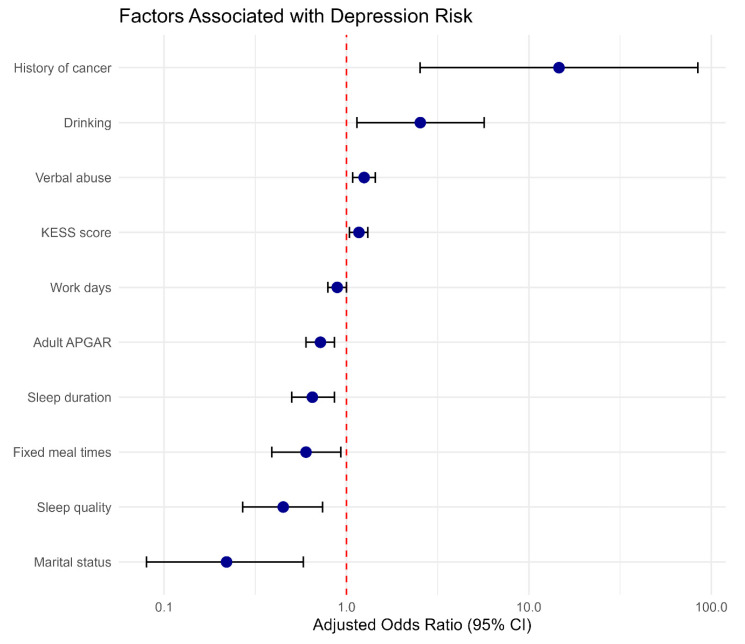
Forest plot of the multivariable logistic regression analysis for depression. The plot presents AORs with their corresponding 95% CIs for variables significantly associated with depression (*p* < 0.05). The vertical dashed line represents an odds ratio of 1.0; estimates to the right indicate increased odds of depression, whereas those to the left indicate protective factors. AOR: adjusted odds ratio, CI: confidence interval, KESS score: Korean Epworth Sleepiness Scale.

In the multivariable logistic regression model, depression (PHQ-9 ≥10) remained independently associated with several demographic, behavioral, psychosocial, and work-related factors after adjustment for potential confounders (Table 4 and Figure 1).

Marital status demonstrated a significant protective association, with married participants having lower odds of depression than those who were unmarried or separated (adjusted OR, 0.22; 95% CI, 0.08–0.58; *p* = 0.002). Among medical comorbidities, a history of cancer was strongly associated with higher odds of depression (adjusted OR, 14.63; 95% CI, 2.53–84.61; *p* = 0.003), whereas hypertension and cardiovascular disease were not independently associated with depression in the fully adjusted model.

Regarding health-related behaviors, current alcohol consumption was significantly associated with increased odds of depression (adjusted OR, 2.54; 95% CI, 1.14–5.68; *p* = 0.023), whereas smoking was not significantly associated. Workload indicators showed limited associations; however, a greater number of working days per month was modestly associated with lower odds of depression (adjusted OR, 0.89 per additional day; 95% CI, 0.79–1.00; *p* = 0.047). The number of night shifts and perceived work intensity relative to other departments were not significant.

Eating-related factors demonstrated a protective effect. Availability of fixed mealtimes was independently associated with lower odds of depression (adjusted OR, 0.60; 95% CI, 0.39–0.93; *p* = 0.024), while perceived sufficiency of mealtime and the ability to take meals despite ED workload were not significantly associated.

Sleep-related variables showed robust and consistent associations. Longer sleep duration was associated with reduced odds of depression (adjusted OR, 0.65; 95% CI, 0.50–0.86; *p* = 0.002), and better subjective sleep quality was also independently protective (adjusted OR, 0.45; 95% CI, 0.27–0.74; *p* = 0.001). In contrast, greater daytime sleepiness, as measured by the Korean Epworth Sleepiness Scale, was associated with increased odds of depression (adjusted OR, 1.17 per one-point increase; 95% CI, 1.04–1.31; *p* = 0.008).

Psychosocial factors remained important. Higher Adult APGAR scores were associated with lower odds of depression (adjusted OR, 0.72 per one-point increase; 95% CI, 0.60–0.86; *p* < 0.001). Exposure to verbal abuse over the past 12 months was independently associated with increased odds of depression (adjusted OR, 1.25 per incident; 95% CI, 1.08–1.44; *p* = 0.002). Perceived safety in the ED showed a trend toward association but did not reach statistical significance after adjustment (adjusted OR, 1.65; 95% CI, 0.95–2.85; *p* = 0.073).

## 4. Discussion

This study was designed to estimate the prevalence of screening positive for depressive symptoms and to identify factors associated with depressive symptoms among emergency physicians in South Korea during a period of substantial healthcare system disruption. In this nationwide survey, 14.9% of participants screened positive for moderate-to-severe depressive symptoms. Our findings indicate that depressive symptoms were associated with multiple underlying medical conditions (including serious illnesses such as cancer), work-related stressors, exposure to violence, sleep-related factors, and psychosocial resources. Patient-related factors, such as poor compliance, verbal abuse, and perceived lack of safety in the ED, emerged as key occupational drivers of depression, even after controlling for age, sex, comorbidities, and workload indicators. These findings align with previous studies showing that workplace violence and moral distress are strongly associated with mental health problems among ED workers [7,17]. Workplace violence and moral distress are strongly associated with mental health problems across the entire spectrum of emergency medical staff, including nurses and paramedics [18]. By quantifying these impacts in a multivariable model, our study reinforces the evidence that the safety and organizational culture of the ED are fundamental determinants of well-being for all frontline medical providers. This shared vulnerability suggests that interventions to mitigate depression must be inclusive and system-wide, addressing the safety of the entire emergency medical team.

Several factors were associated with lower odds of depressive symptoms. The multivariable logistic regression analysis demonstrated that being married, maintaining regular mealtimes, and having a higher overall wellness status (Adult APGAR score) were associated with significantly lower odds of screening positive for depressive symptoms. Notably, both longer sleep duration and better sleep quality were protective against depressive symptoms. These findings align with previous domestic studies highlighting the critical link between sleep–wake disorders and mental health among Korean emergency medicine physicians and residents [4,12,19,20]. Furthermore, the roles of lifestyle regularity and perceived support are consistent with international findings, which identify barriers to healthy sleep and social isolation as major drivers of physician distress [21]. These results highlight the importance of both organizational-level interventions (e.g., improving staff allocation, strengthening safety policies, and addressing patient–physician conflict) and individual-level strategies (e.g., sleep hygiene, structured meal breaks, and strengthening family and social support) in mitigating depression among emergency physicians. Additionally, the strong association between a history of cancer and depression underscores the need to pay particular attention to physicians with serious medical conditions. Such individuals may experience a double burden of personal illness and professional stress, necessitating tailored institutional support and occupational health monitoring.

The strong association between a history of cancer and depressive symptoms should be interpreted cautiously. Physicians with a prior cancer diagnosis may experience ongoing psychological vulnerability related to fear of recurrence, treatment sequelae, and health-related anxiety [22]. However, the number of participants reporting a history of cancer was relatively small, and the wide confidence interval suggests possible statistical instability. Small subgroup effects and residual confounding cannot be excluded, as detailed clinical information (e.g., cancer type, stage, and treatment history) was not available. Longitudinal studies with more granular clinical data are needed to clarify this association.

An additional finding that warrants careful interpretation is the inverse association between the number of working days per month and depressive symptoms. Although this result may appear counterintuitive, several explanations should be considered. First, given the cross-sectional design, reverse causation cannot be excluded; physicians experiencing depressive symptoms may have reduced their clinical workload, resulting in fewer working days per month. Second, a higher number of working days may reflect greater professional engagement, organizational integration, or perceived role stability, which could be associated with better psychological resilience. Therefore, this association should be interpreted cautiously, and longitudinal studies are needed to clarify the temporal relationship between workload and depressive symptoms.

The prevalence of depressive symptoms among emergency physicians in this study was 14.9%, which is lower than the 27.0% reported in a previous study [12]. This downward trend mirrors observations in the general population, where the prevalence of depressive symptoms has declined gradually following the end of pandemic-related social restrictions. This difference likely reflects the distinct time points at which the studies were conducted; while the prior study reflected the severe burnout experienced during the peak of the COVID-19 pandemic, the current study reflects a post-pandemic stabilization phase. Nevertheless, the prevalence of 14.9% remains more than double that found in the general population (approximately 6–7% according to the Korea National Health and Nutrition Examination Survey, 2023) [2]. This finding confirms that despite the overall post-pandemic recovery, emergency physicians continue to represent a high-risk group for depression, facing persistent occupational stressors that exceed those of the general public [23].

These findings are reinforced by international literature, confirming that the psychological vulnerability of emergency physicians is a global phenomenon. Shanafelt et al. [24] demonstrated that emergency medicine consistently ranks among specialties with the highest burnout rates and lowest satisfaction with work–life integration in the United States. Their research underscores that mental health challenges in this field are not transient but represent a persistent, structural hazard. Furthermore, Stehman et al. [25] noted that drivers of physician distress are diverse, ranging from environmental stressors such as overcrowding to interpersonal violence. Although high stress is a universal hallmark of emergency medicine, the specific determinants are shaped by socio-legal environments. In South Korea, interpersonal hostility and safety concerns emerge as the primary occupational stressors undermining physician well-being, potentially more so than administrative burdens often cited in Western counterparts.

Although certain contextual factors—such as the recent healthcare system disruption and medico-legal environment—may reflect features specific to South Korea, the core determinants identified in this study are supported by a substantial body of international literature. In particular, the associations between mental health outcomes and sleep disruption, workplace violence, and chronic medical conditions (e.g., cancer) have been observed across diverse healthcare systems [20,22,26,27]. Therefore, while the magnitude and contextual expression of these factors may vary by country, the overall pattern of findings is likely generalizable beyond the Korean setting. As such, this study may contribute meaningful evidence to the global discourse on emergency physicians’ mental health.

Finally, the significant association between exposure to violence and depressive symptoms persists despite increased legal penalties for interfering with emergency medical services. This finding underscores the limitations of current legislative efforts. While amendments to the Emergency Medical Service Act and strengthened penalties are steps in the right direction, they remain insufficient. More comprehensive social and institutional changes—such as enhanced on-site security and a cultural shift in patient-provider interactions—are imperative to protect the mental health of emergency physicians.

### 4.1. Clinical Implications

The present findings have important clinical and organizational implications. Given the multifactorial determinants identified, interventions should extend beyond individual resilience training to include structural reforms addressing workplace safety, staffing stability, and workload distribution. Routine screening for depressive symptoms—particularly among physicians with chronic medical conditions or high exposure to workplace violence—may facilitate early identification and timely support. Protecting the mental health of emergency physicians is essential not only for individual well-being but also for patient safety and healthcare system resilience.

At the organizational level, tailored scheduling policies that prioritize the preservation of sleep quality and the reduction in cumulative fatigue are warranted. Strategies such as limiting consecutive night shifts, ensuring adequate recovery time between shifts, and systematically monitoring overall workload intensity may help mitigate chronic sleep disruption and psychological strain [26]. In parallel, legal and institutional mechanisms designed to protect healthcare workers from workplace violence in the emergency department should be strengthened and consistently enforced [27]. Ultimately, sustainable improvement in physicians’ mental health requires integrated, system-level interventions that balance operational demands with structured opportunities for recovery and psychological safety within the emergency department. Such measures may require coordinated efforts at both the hospital administration and national policy levels to ensure long-term feasibility and effectiveness.

### 4.2. Strengths and Limitations

This study has several strengths. First, it is based on a nationwide survey conducted at five-year intervals, allowing timely assessment during a unique period of healthcare system disruption. Second, the inclusion of environmental, socio-legal, and individual health factors—including chronic medical conditions—enabled a comprehensive evaluation of determinants of depressive symptoms. Third, validated instruments with good internal consistency were used, enhancing measurement reliability.

This study has some limitations that must be acknowledged. First, as a cross-sectional study, the survey was conducted at a single point in time. While this design allows identification of associations, it does not establish causality. In addition, the temporal sequence between depressive symptoms and associated factors cannot be determined; therefore, directionality of the observed associations cannot be inferred.

Second, the study relied on self-reported measures of subjective factors such as the PHQ-9 and Adult APGAR scores. Consequently, there is a possibility of over- or underestimation based on the respondents’ emotional state at the time of the survey. This methodology may also introduce social desirability bias, particularly concerning reluctance to accurately report mental health issues within the medical profession.

Third, the findings are limited by representativeness and selection bias. The sample was not drawn from the entire population of emergency physicians but consisted of individuals who voluntarily participated in the survey. This voluntary participation may introduce non-response bias, as physicians experiencing high workloads may not have had time to respond. Conversely, those with a pre-existing interest in mental health topics may have selectively participated. Therefore, generalization to the entire specialist population should be done with caution.

Finally, a different diagnostic threshold for the PHQ-9 score was employed compared with some previous studies. While prior studies often used a cut-off of ≥ 11 to indicate significant depressive symptoms, this study adopted a cut-off of ≥10 [12,28]. This lower threshold may result in the inclusion of individuals who would not have been classified as depressed in previous research, potentially leading to an overestimation of the prevalence of depressive symptoms in this study.

## 5. Conclusions

In this nationwide survey of emergency physicians, depression was common and independently associated with multiple demographic, behavioral, psychosocial, and work environment factors. Work-related burdens, lifestyle irregularity, sleep health, and overall wellness were strongly associated with depression.

Protective factors included being married, regular meal availability, longer sleep duration, better sleep quality, and higher Adult APGAR scores. In contrast, a history of cancer, alcohol consumption, increased daytime sleepiness, and frequent verbal abuse were independently associated with higher odds of depression.

These findings suggest that addressing depression among emergency physicians requires an approach that extends beyond workload reduction and incorporates strategies to improve sleep health, meal regularity, psychosocial support, and workplace safety. A multifaceted, organizational-level approach may be essential for reducing depression and promoting well-being among emergency physicians.

## Figures and Tables

**Table 1:** Baseline characteristics according to depression status.

Characteristic	No Depression *n* = 632	Depression *n* = 111	*p*-Value
Age	43.2 ± 7.9	43.4 ± 7.4	0.774
Sex, *n* (%)			0.462
Female	88 (13.9)	12 (10.8)	
Male	544 (86.1)	99 (89.2)	
Marital status, *n* (%)			0.001
Married	555 (88.4)	85 (76.6)	
Single	73 (11.6)	26 (23.4)	
Type of position, *n* (%)			0.825
Faculty	137 (21.7)	28 (25.2)	
Clinical emergency physician	303 (47.9)	50 (45.0)	
Clinical professor	98 (15.5)	17 (15.3)	
Military service	37 (5.9)	4 (3.6)	
Private clinic	42 (6.6)	8 (7.2)	
Others	15 (2.4)	4(3.6)	
Hypertension, *n* (%)	146 (23.2)	40 (36.4)	0.005
Diabetes, *n* (%)	48 (7.7)	14 (12.8)	0.109
Arrhythmia, *n* (%)	44 (7.1)	20 (18.2)	<0.001
Cardiovascular disease, *n* (%)	17 (2.7)	12 (11.2)	<0.001
Cerebrovascular disease, *n* (%)	10 (1.6)	2 (1.9)	1.000
Cancer, *n* (%)	18 (2.9)	12 (11.1)	<0.001
BMI ≥ 30, *n* (%)	133 (21)	33 (30)	0.052
Exercise frequency (per week)	2.4 ± 1.7	1.9 ± 1.6	0.003
Smoking, *n* (%)	115 (18.2)	27 (24.3)	0.169
Smoking frequency (pack-years)	17.3 ± 24.5	29.4 ± 35.3	0.067
Drinking, *n* (%)	267 (42.3)	50 (45.0)	0.665
Drinking frequency (per month)	6.0 ± 5.3	7.5 ± 5.7	0.035

Data are presented as mean ± standard deviation (SD) for continuous variables and number (percentage) for categorical variables. BMI: body mass index.

**Table 2:** Work environment, workload, and eating-related factors.

Characteristic	No Depression *n* = 632	Depression *n* = 111	*p*-Value
Schedule satisfaction	3.3 ± 0.9	2.8 ± 1.0	<0.001
Salary satisfaction	2.9 ± 1.0	2.7 ± 1.1	0.070
Number of working days per month	10.6 ± 3.4	10.1 ± 3.4	0.272
Number of night shifts per month	5.1 ± 2.0	5.0 ± 1.7	0.765
Total working hours per month	123.4 ± 49.5	123.6 ± 50.4	0.970
Annual ED visits (2024)	26,603 ± 12,973	27,898 ± 13,482	0.096
Number of emergency medicine specialists	9.1 ± 4.2	9.1 ± 3.9	0.944
Number of emergency medicine residents	1.1 ± 1.7	1.7 ± 2.9	0.179
Number of interns	0.4 ± 1.0	0.5 ± 1.1	0.483
Perceived work-hour burden vs. other departments	3.1 ± 0.9	3.3 ± 0.9	0.042
Perceived work intensity vs. other departments	3.8 ± 0.8	4.3 ± 0.8	<0.001
Salary adequacy relative to work intensity	2.8 ± 0.7	2.5 ± 0.8	<0.001
Burden of patient volume	3.3 ± 0.8	3.7 ± 1.0	0.001
Burden of critical care management	3.4 ± 0.9	4.0 ± 1.0	<0.001
Burden of explaining clinical decisions to other departments	3.2 ± 1.0	3.7 ± 1.1	<0.001
Burden due to the attitudes of other departments toward emergency medicine	3.4 ± 1.0	4.0 ± 0.9	<0.001
Burden of patient transfer-out	4.1 ± 1.2	4.5 ± 1.0	0.001
Burden of accepting transferred patients	3.4 ± 1.1	3.7 ± 1.2	0.010
Burden of admission requests and discharge refusals	3.2 ± 1.1	3.6 ± 1.2	0.001
Burden of relationships with tertiary hospitals	3.1 ± 1.1	3.4 ± 1.2	0.013
Burden of relationships with long-term care facilities and vulnerable institutions	3.3 ± 1.1	3.7 ± 1.2	0.004
Burden of relationships with public health authorities	2.8 ± 1.1	3.3 ± 1.4	0.003
Burden of the physician shortage	3.5 ± 1.1	3.9 ± 1.2	0.004
Burden of the nursing staff shortage	3.3 ± 1.1	3.6 ± 1.1	0.022
Burden of the emergency medical technician (EMT) shortage	3.0 ± 1.2	3.3 ± 1.2	0.098
Burden of patient non-compliance	3.2 ± 1.0	3.7 ± 0.8	<0.001
Burden related to security staffing	2.8 ± 1.2	2.9 ± 1.2	0.672
Burden related to prehospital emergency medical services and patient transport	3.8 ± 1.0	4.1 ± 0.9	0.003
Burden of relationships among emergency physicians	1.9 ± 1.0	2.3 ± 1.1	0.001
Burden of relationships with emergency nurses and EMTs	1.9 ± 0.9	2.1 ± 1.1	0.031
Burden related to work scheduling	3.0 ± 1.0	3.4 ± 1.1	<0.001
Availability of fixed mealtimes	3.2 ± 1.2	2.5 ± 1.2	<0.001
Ability to take meals despite the ED workload	2.5 ± 1.3	1.9 ± 1.2	<0.001
Access to regular hospital meals	2.8 ± 1.3	2.3 ± 1.3	0.001
Perceived sufficiency of mealtime	2.4 ± 1.3	1.8 ± 1.2	<0.001
Regular breakfast consumption	2.5 ± 1.4	2.4 ± 1.3	0.184
Night-time meal consumption during night shifts	2.3 ± 1.2	2.6 ± 1.3	0.037
Perceived dietary healthiness	2.8 ± 1.0	2.0 ± 0.9	<0.001

Data are presented as mean ± standard deviation (SD) for continuous variables and number (percentage) for categorical variables. ED: emergency department, EMT: emergency medical technician.

**Table 3:** Mental health, resilience, violence, and legal issues.

Characteristic	No Depression *n* = 632	Depression *n* = 111	*p*-Value
Self-rated health status	3.2 ± 0.9	2.4 ± 0.8	<0.001
Sleep duration (hours/day)	7.4 ± 1.2	7.0 ± 1.7	0.018
Sleep quality	2.8 ± 1.0	2.0 ± 0.8	<0.001
Korean Epworth Sleepiness Scale	4.2 ± 3.0	7.0 ± 3.7	<0.001
Adult APGAR Score (wellness assessment tool)	5.3 ± 2.4	3.0 ± 2.1	<0.001
Number of verbal abuse incidents (past 12 months)	2.9 ± 3.1	5.4 ± 3.5	<0.001
Number of physical assault incidents (past 12 months)	0.2 ± 1.0	0.6 ± 1.2	0.009
Verbal abuse in the ED (past 1 month)	2.3 ± 2.9	3.5 ± 3.4	0.002
Physical assault in the ED (past 1 month)	0.3 ± 1.1	0.6 ± 1.6	0.038
Perceived safety in the ED	3.1 ± 0.9	2.8 ± 0.9	0.002
Recent legal dispute (past 12 months), *n* (%)	154 (31)	42 (48)	0.003
Civil or criminal litigation experience, *n* (%)	166 (33)	39 (44)	0.056

Data are presented as mean ± standard deviation (SD) for continuous variables and number (percentage) for categorical variables. ED: emergency department.

**Table 4:** Multivariable logistic regression analysis for depression.

Variable	Adjusted OR	95% CI	*p*-Value
Marital status (Married vs. unmarried/separated)	0.22	(0.08–0.58)	0.002
History of hypertension diagnosis (yes vs. no)	1.24	(0.54–2.88)	0.611
History of cardiovascular disease diagnosis (yes vs. no)	3.87	(0.64–23.48)	0.142
History of cancer diagnosis (yes vs. no)	14.63	(2.53–84.61)	0.003
Smoking (yes vs. no)	1.71	(0.71–4.13)	0.233
Drinking (yes vs. no)	2.54	(1.14–5.68)	0.023
Number of working days per month (per 1-day increase)	0.89	(0.79–1)	0.047
Number of night shifts per month (per 1-day increase)	0.91	(0.74–1.13)	0.398
Perceived work intensity vs. other departments	1.46	(0.86–2.47)	0.160
Burden of critical care management	1.10	(0.72–1.69)	0.647
Burden of patient non-compliance	1.16	(0.71–1.91)	0.547
Burden related to security staffing	0.76	(0.54–1.08)	0.131
Burden of relationships among emergency physicians	1.38	(0.94–2.03)	0.103
Availability of fixed mealtimes (higher = more regular)	0.60	(0.39–0.93)	0.024
Ability to take meals despite the ED workload	0.91	(0.56–1.47)	0.693
Perceived sufficiency of mealtime	0.78	(0.47–1.3)	0.341
Self-rated health status (higher = better)	0.61	(0.36–1.05)	0.075
Sleep duration (hours/day)	0.65	(0.5–0.86)	0.002
Sleep quality (higher = better)	0.45	(0.27–0.74)	0.001
Korean Epworth Sleepiness Scale (higher = worse)	1.17	(1.04–1.31)	0.008
Adult APGAR score (higher = better functionality)	0.72	(0.6–0.86)	<0.001
Number of verbal abuse incidents (past 12 months)	1.25	(1.08–1.44)	0.002
Perceived safety in the ED (higher = safer)	1.65	(0.95–2.85)	0.073

ED: emergency department, OR: odds ratio, CI: confidence interval.

## Data Availability

The data presented in this study are available from the corresponding author upon reasonable request.

## Abbreviations

The following abbreviations are used in this manuscript:

ED	Emergency department
KEPS	Korean emergency physician survey
AORs	Adjusted odds ratios
CI	Confidence interval
EMT	Emergency medical technician
BMI	Body mass index
